# Molecular Mechanisms by Which Linear Versus Branched Alkyl Chains in Nonionic Surfactants Govern the Wettability of Long-Flame Coal

**DOI:** 10.3390/molecules30244686

**Published:** 2025-12-07

**Authors:** Boyu Li, Guochao Yan, Shaoqi Kong, Kuangkuang Wu, Yanheng Wang

**Affiliations:** College of Mining Engineering, Taiyuan University of Technology, Taiyuan 030024, China; 2023521218@link.tyut.edu.cn (B.L.); 2023510801@link.tyut.edu.cn (K.W.); 2023521279@link.tyut.edu.cn (Y.W.)

**Keywords:** nonionic surfactant, long-flam coal, wettability

## Abstract

Improving the wettability of coal dust with nonionic surfactants is crucial for mitigating environmental pollution. Here we compare two nonionic surfactants with distinct architectures—n-octyl-α-D-glucoside (OG) and Isooctyl glucoside (APG08)—to dissect how linear versus branched C8 chains govern the wetting of long-flame bituminous coal dust. Sedimentation and contact-angle measurements show that the linear OG, with reduced steric hindrance, assembles into a denser interfacial layer and delivers superior wetting. Corroborating spectroscopic and microscopic analyses (FTIR, XPS, and SEM) reveal that OG treatment increases hydroxyl functionalities and the O-element fraction at the coal surface; OG also drives stronger particle aggregation, consistent with markedly enhanced adsorption on coal. Molecular dynamics simulations further indicate tighter OG adsorption, a more homogeneous coal–water interfacial structure, and stronger binding of water to OG-modified surfaces. Collectively, these results identify chain linearity as a key design lever for nonionic glucosides and establish OG as a more effective wettability promoter for long-flame coal dust.

## 1. Introduction

Coal is abundant and accounts for a substantial share of global energy consumption. Yet dust is generated at every stage of mining, posing serious risks to human health. Fine particles remain suspended in enclosed workspaces and, with chronic inhalation, gradually deposit in the lungs, causing coal workers’ pneumoconiosis [1,2,3,4,5]. Effective dust control and management, together with appropriate safety protocols, are therefore pivotal for the mining sector [6,7,8,9].

In recent years, ventilation with dust extraction, in-seam water injection, spray-based suppression, air-curtain isolation and foam dusting have emerged as the principal strategies to curb particulate levels in coal production [10,11,12,13,14]. A complementary approach—adding surfactants to water to lower surface tension and enhance coal-dust wettability—has shown notable success [15,16,17]. Surfactant additives have been reported to accelerate the settling of coal-dust agglomerates [18]. Bai et al. [19] examined the molecular mechanisms by which anionic and cationic surfactants improve the wettability of anthracite coal, comparing adsorption strengths and wetting enhancement. Geng et al. [20] studied mixtures of amphoteric surfactants for hydrophobic modification of lignite and suppression of liquid readsorption. Liu et al. [21] used surface tension and contact angle analyses to determine that a mass ratio of 3:1 between sodium dodecyl sulfate and 1-butyl-3-methylimidazolium tetrafluoroborate is optimal for wetting, clarifying the interactions between wetting agents and coal. Wang et al. [22] conducted experiments with six silicone-based surfactants and, by measuring wetting time, contact angle and surface tension, identified polyoxymethylene-modified heptamethyltrisiloxane as the most effective wetting agent. Wang et al. [23] proposed a paired-surfactant formulation and evaluated its wettability using surface tension, contact angle, and coal dust sedimentation; relative to untreated water, a blend containing 0.025 wt% fatty-acid methyl-ester ether oxide and 0.025 wt% coconut diethanolamide delivered the best wetting performance. Thompson et al. [24] reported pronounced interactions between proteins and the surfactant N,N-dimethyldodecylamine oxide (OA-12) that alter protein stability and activity; the structure of protein–surfactant complexes also modulates interfacial activity, thereby improving water’s wetting capability.

Despite this progress, the influence of linear versus branched architectures in alkyl-glucoside nonionic surfactants on the surface wetting of long-flame coal remains underexplored. Here, we combine molecular dynamics (MD) simulations and theoretical analysis with sedimentation tests, contact-angle measurements, and XPS, FTIR, and SEM characterization to compare n-octyl-α-D-glucoside (OG) with isooctyl glucoside (APG08). From multiple vantage points, we delineate how chain topology—linear versus branched—governs interfacial adsorption and wetting, and we identify the mechanistic origins of the wettability contrast observed on long-flame coal. This work provides a theoretical foundation for the design of high-efficiency dust suppressants and offers practical guidance for structure–function recognition in alkyl-glucoside surfactants.

## 2. Experiments and Simulations

### 2.1. Experimental Materials

Long-flame coal is inherently poorly wettable. In this study, we used a long-flame coal collected from the Shangwan mine of the Shenhua Shendong Coal Group (Table 1). The sample was crushed, sieved to 200 mesh, and the resulting dust was acid-washed and deashed with HCl and HF. The treated coal was then dried at 398 K for 10 h to complete dehydration and stored for subsequent experiments [25,26].

**Table 1:** Basic analysis of long-flame coal.

Sample	Industrial Analysis (wt%)			Density (g/cm^3^)	Elemental Analysis (wt%, d.m.m.f)				
	M_ad_	A_ad_	V_daf_		C	H	N	S	O
long-flame coal	2.55	1.25	37.77	1.26	78.46	4.52	1.18	0.75	15.09

We examined two nonionic surfactants—n-octyl-α-D-glucoside (OG) and isooctyl glucoside (APG08), both purchased from Shanghai Macklin Biochemical Co., Ltd., Shanghai, China (Table 2)—each with analytical purity ≥ 98%. Ultrapure water was used as the solvent in all experiments. Surfactant solutions and water were sonicated for 5 min to accelerate dissolution, then stirred on a thermostated magnetic shaker at 298.15 K and 2000 rpm for 12 h to ensure complete dissolution [27,28]. Working solutions were prepared at 1300 mg L^−1^, a concentration above the respective critical micelle concentration (CMC) [29].

### 2.2. Adsorption Experiment

Coal–surfactant adsorption: In a 500 mL beaker, 400 mg of acid-washed coal was mixed with 400 mL of a series of surfactant solutions. The suspension was stirred on a thermostated magnetic stirrer at 298.15 K and 800 rpm for 12 h to ensure adsorption. After equilibration, the mixture was centrifuged at 4000 rpm for 20 min to separate the coal from the supernatant [30,31,32,33]. The coal pellet was collected, rinsed thoroughly with ultrapure water to remove residual surfactant, and vacuum-filtered three times to complete the separation. The cleaned coal was then dried and sealed for subsequent use.

### 2.3. Surface Characterization

Coal-dust settling time was used as a macroscopic indicator of dispersibility and wetting rate. Experiments were conducted in graduated cylinders (inner diameter 20 mm, height 80 mm). Aliquots (50 mL) of OG solution, APG08 solution and ultrapure water (control) were placed in separate vessels, and the settling time was defined as the duration required for all particles to reach the bottom. Each test was performed in triplicate, and the mean settling time was reported [34].

Contact angle was used as a quantitative proxy for surfactant-enhanced wettability. Measurements were performed on a JY-82C (Chengde Testing Equipment Co., Ltd., Chengde, China) goniometer equipped with a video frame-capture system to record dynamic wetting. Tests employed ultrapure water, OG and APG08 solutions at identical concentrations, and untreated coal as a reference. For each trial, the droplet volume was fixed at 0.5 μL. Coal powders were pressed into circular pellets (~12 mm diameter, ~2 mm thickness) using an IR press at 20 MPa for 5 min [35,36]. Images were processed in ImageJ (Version:1.54g) to improve data fidelity. Each condition was measured in triplicate and averaged [37].

### 2.4. Coal Structural Characterization

The FTIR experiments were conducted using the Thermo Scientific Nicolet iS20 Fourier Transform Infrared Spectrophotometer (Thermo Fisher Scientific, Waltham, MA, USA). FTIR spectra were recorded to quantify changes in surface functional groups following modification with OG and APG08. Prior to analysis, coal samples were mixed with KBr at a 1:100 mass ratio and vacuum-dried to remove moisture. Transparent pellets were then pressed at 10 MPa [38,39]. Spectra were collected over 4000–400 cm^−1^ with a mirror velocity of 0.4747 cm s^−1^, 32 scans and a resolution of 4 cm^−1^ [40]. Peak fitting was performed in Origin using the peak-analysis module [41].

XPS experiments were performed using the Thermo Scientific K-Alpha X-ray electron spectrometer (Thermo Fisher Scientific, USA). X-ray photoelectron spectroscopy (XPS) was used to determine the surface distributions of carbon (C) and oxygen (O) in OG- and APG08-modified coal. Measurements employed a monochromatic Al Kα source (hν = 1486.6 eV), with analysis-chamber pressure better than 5.0 × 10^−7^ mbar during acquisition. Survey spectra were recorded at a pass energy of 100 eV with 1 eV energy steps [42]. High-resolution (narrow) scans were accumulated over five cycles (element-dependent dwell times) at a pass energy of 50 eV and a step size of 0.05 eV. Peak fitting and data processing were performed using Avantage (Version:6.9.0) [26,43].

The TESCAN MIRALMS scanning electron microscope (TESCAN ORSAY HOLDING, a. s., Kohoutovice, Czech Republic) was used to characterize microstructural changes in coal before and after surfactant adsorption, with emphasis on pore architecture and surface agglomerate distributions to elucidate fundamental textural properties [44].

### 2.5. Molecular Simulation

**Figure 1:**
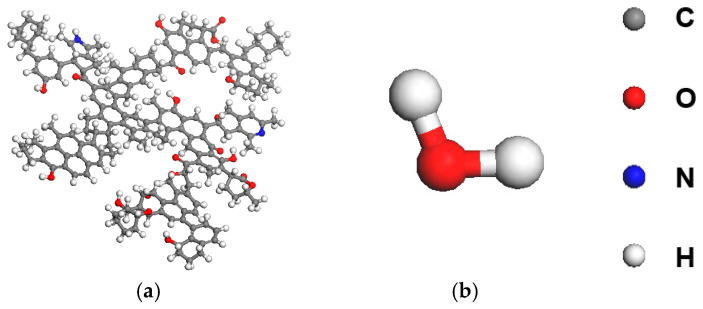
Molecular models: (**a**) Coal; (**b**) H_2_O.

Molecular models of water, long-flame coal, APG08 and OG were constructed in Materials Studio as shown in Figure 1. Representative structures were constructed using the Shendong long-flame coal framework to detect microscopic adsorption. Molecular dynamics (MD) simulations were performed with the Forcite module using the COMPASS III force field in the NVT ensemble. Each simulation cell comprised a coal layer assembled from 10 long-flame coal models, a surfactant layer of 20 molecules and a water layer of 2000 molecules; a 50 Å vacuum slab was included to suppress mirror artifacts. Systems underwent 100 ps of geometry optimization with the Smart algorithm for energy minimization, followed by production runs of 1,000,000 steps with a 1 fs timestep (total 1000 ps). Post-processing included calculations of contact surface area and interaction energies, analysis of mass-density profiles, water mean-squared displacement and hydrogen-bond counts, thereby corroborating the experimental findings [45,46,47].

## 3. Results and Discussion

### 3.1. Surface Characterization Analysis

#### 3.1.1. Settlement Experimental Analysis

**Figure 2:**
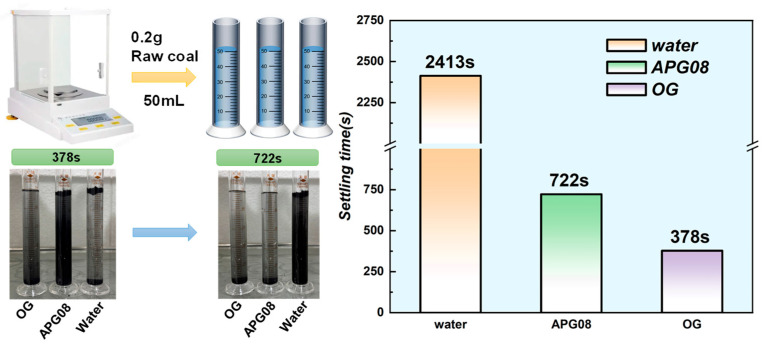
Settlement experiment flow chart and settlement time diagram.

Wettability was assessed by the settlement test, wherein shorter settling times indicate stronger surfactant-mediated wetting of coal dust. To quantify these observations, the settling time t (s) for each medium was determined as follows. For each test, an accurately weighed amount of coal dust (0.2 g) was dispersed in a fixed volume(50 mL) of ultrapure water or surfactant solution in a graduated cylinder. Immediately after homogenization of the coal–liquid dispersion by manual inversion (taken as t = 0), a stopwatch was started. The measurement was stopped when a stable solid–liquid interface formed, with no visibly suspended or floating particles remaining in the bulk liquid and the height of the settled coal layer remaining unchanged for at least 30 s. Each condition was tested in triplicate. The settling times of long-flame coal dust in different media are shown in Figure 2. After 378 s, all particles in the OG solution had settled, whereas the APG08 dispersion remained black, and the water control showed only partial settling with a semitransparent supernatant. By 722 s, complete settling was also observed for APG08, and more particles had settled in the water. Full sedimentation in water occurred at 2413 s. These macroscopic observations demonstrate that OG confers markedly superior wettability relative to APG08.

#### 3.1.2. Contact Angle Analysis

**Figure 3:**
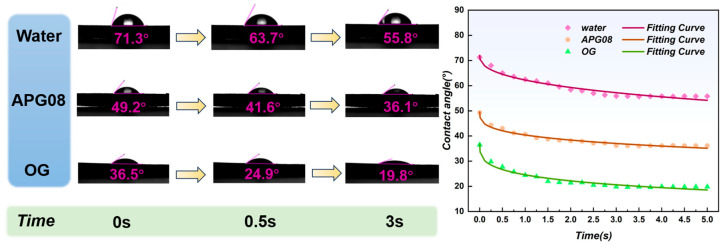
Dynamic contact angle changes of water, APG08, and OG.

Figure 3 shows the time evolution of the contact angle on long-flame coal powder in ultrapure water, APG08 and OG solutions. At t = 0, the initial contact angles are 71.3°, 49.2° and 36.5° for water, APG08 and OG, respectively, confirming that both alkyl glycoside surfactants markedly improve the instantaneous wetting of the coal surface compared with ultrapure water. As time increases, the contact angles decay rapidly within the first few seconds and then approach quasi-equilibrium values. Among the three liquids, OG exhibits the lowest final contact angle (19.8°) and reaches equilibrium within approximately 3 s, whereas APG08 and water show higher equilibrium values and slower relaxation. These trends are consistent with the settling experiments, in which OG also leads to the shortest settling time of coal particles.

To quantify the wetting kinetics, the *θ*(*t*) curves in Figure 3 were fitted using the semi-empirical model given by Equation (1), which has been widely applied to describe the kinetic evolution of contact angles on porous and heterogeneous substrates [48]:

(1) $$\theta \left(t\right)=\theta_{i}\times \exp\left(-k_{h}t^{n}\right)$$

The fitted initial contact angles *θ_i_*, kinetic constants *k_h_*, exponents *n* and coefficients of determination (R^2^) are summarized in Table 3. All three systems are well captured by the model, with R^2^ values of 0.9587, 0.9743 and 0.9662 for water, APG08 and OG, respectively, indicating that Equation (1) provides an adequate description of the contact angle decay on long-flame coal within the 0–5 s time window. The kinetic constant *k_h_* clearly discriminates the three liquids. The values follow the order: *k_h_*(OG) = 0.4727 s^−n^ > *k_h_*(APG08) = 0.2039 s^−n^ > *k_h_*(water) = 0.1447 s^−n^, demonstrating that both surfactant solutions accelerate the wetting process compared with ultrapure water, and that OG produces the fastest relaxation of the contact angle. This kinetic behavior is fully consistent with the reduced equilibrium contact angle and shortened settling time observed for OG at the macroscopic scale, confirming that the linear C8 alkyl chain of OG is more effective in promoting rapid coal wetting than the branched C8 chain of APG08.

The exponent n lies between 0 and 1 for all systems (0.4179 for water, 0.3239 for APG08 and 0.2093 for OG), which is characteristic of a stretched-exponential decay arising from a combination of interfacial spreading and liquid absorption into the porous coal matrix. The relatively larger n value for water suggests a more homogeneous, absorption-dominated wetting process on the pristine hydrophobic coal surface. In contrast, the lower n values for APG08 and, in particular, OG indicate a stronger contribution of rapid interfacial spreading enabled by the adsorbed surfactant layers.

### 3.2. Coal Structural Characterization Analysis

#### 3.2.1. XPS Analysis

X-ray photoelectron spectroscopy (XPS) was used to quantify near-surface composition within the relevant binding-energy window, with particular attention to oxygenated functionalities (–OH, –COOH, C=O) that increase surface polarity and hydrogen-bonding with water, thereby enhancing adsorption. To assess how n-octyl-α-D-glucoside (OG) and isooctyl glucoside (APG08) regulate the long-flame coal surface, we acquired survey spectra and performed peak deconvolution to track the distributions of C and O and the evolution of functional groups in raw versus modified coal. Marked differences in C and O contents emerged after adsorption of the two nonionic surfactants [49].

**Figure 4:**
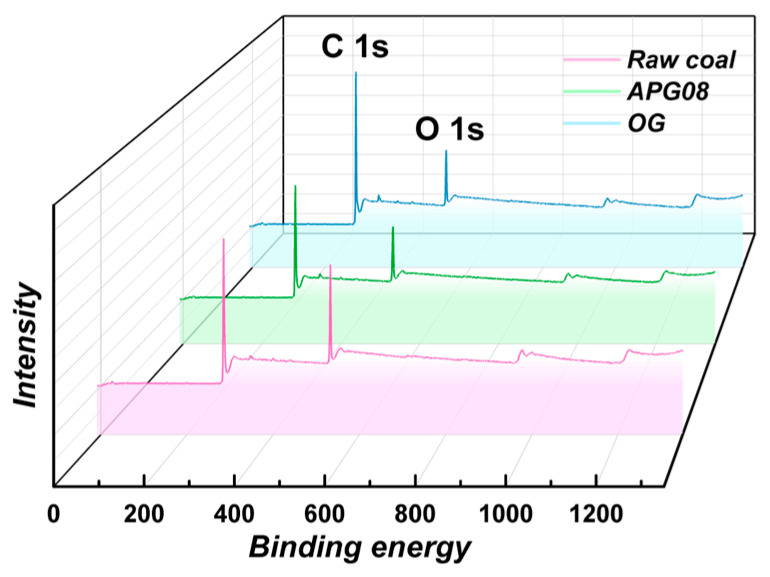
XPS spectrum of long-flame coal.

**Figure 5:**
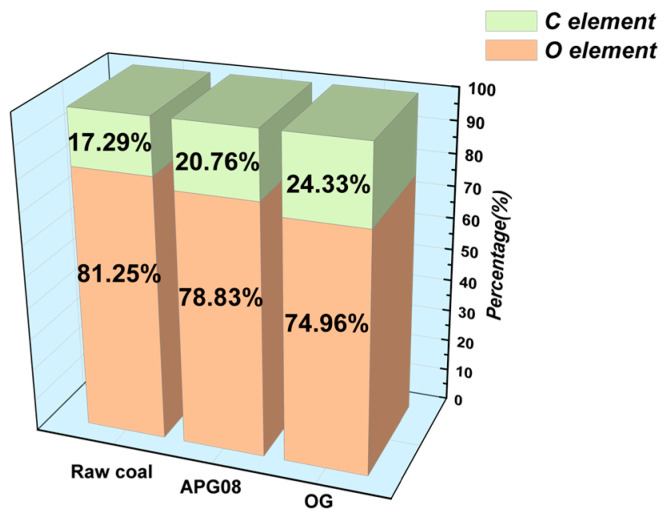
Percentage of C and O elements in three coal samples.

Survey spectra (Figure 4) show that the principal changes between raw and treated samples reside in the C 1s and O 1s envelopes, indicating that surfactant adsorption directly alters the relative abundances of C and O at the interface. Quantitative analysis (Figure 5) further reveals that, after OG treatment, the C 1s fraction decreases from 81.25% to 74.96%, while O 1s increases from 17.29% to 24.33%; the O/C ratio accordingly rises from 0.21 to 0.32. These shifts indicate that surfactant molecules adsorb onto the coal dust, attenuating carbon-rich functionalities and enriching oxygenated groups at the surface. The resulting increase in hydrophilic head-group exposure enhances surface hydrophilicity, improves wettability and promotes water uptake by the dust. By comparison, APG08 produces smaller changes (C 1s −2.42%, O 1s +3.47%), underscoring the stronger adsorption of OG on coal and indicating that the observed improvements in wettability primarily arise from surfactant adsorption [50].

**Figure 6:**
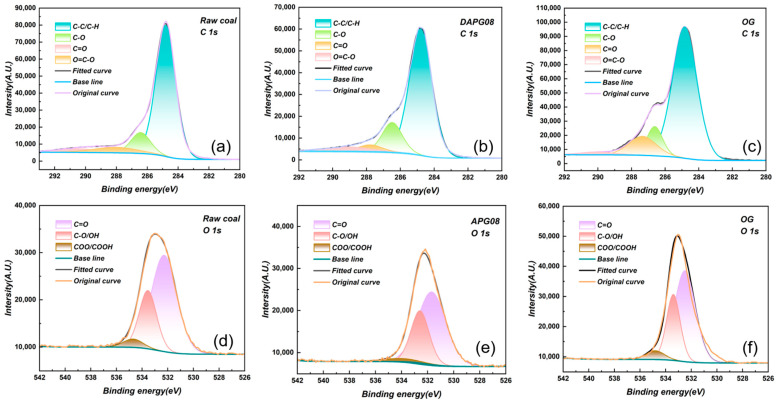
Fitting of C1s and O1s peaks for Raw coal, APG08 modified coal, and OG modified coal: (**a**) C 1s fitted peak of raw coal; (**b**) C 1s fitted peak of APG08-modified coal; (**c**) C 1s fitted peak of OG-modified coal; (**d**) O 1s fitted peak of raw coal; (**e**) O 1s fitted peak of APG08-modified coal; (**f**) O 1s fitted peak of OG-modified coal.

To resolve the chemical states of surface carbon after surfactant-assisted wetting, we performed peak-by-peak fitting on the C 1s peak. (Figure 6a–c). The dominant hydrophobic component (C–C/C–H)—weakly polar owing to the similar electronegativities of C and H—renders the raw coal surface hydrophobic, comprising 76.01% in the untreated sample in Table 4. After modification, this fraction decreases to 73.60% with OG and to 70.44% with APG08, directly diminishing the surface’s repulsion toward water. By contrast, polar, hydrophilic moieties (C–O and C=O), enabled by the high electronegativity of oxygen and capable of hydrogen bonding with water, rise markedly upon treatment: for OG, C–O increases from 12.04% to 19.62%, whereas APG08 yields 18.19%, further reinforcing surface hydrophilicity. Thus, both surfactants promote oxygenated functionalities—likely by adsorption that exposes additional C–O groups or forms new C–O bonds—yet the effect is more pronounced for OG. Peak-to-peak fitting of the O 1s peak region (Figure 6d–f) captures the finer evolution of oxygen chemistries, revealing OH/C–O, C=O and COO/COOH components on raw and modified surfaces alike [51]. Notably, in Table 4, carboxyl content surges after treatment: from 3.29% in raw coal to 4.21% with APG08 and to 7.15% with OG, the latter being 2.17-fold higher than the untreated value. As the most strongly polar O-bearing group, –COOH can dissociate to –COO^−^, which engages water via bifurcated hydrogen bonding (–COO^−^···H–O–H), rationalizing the superior hydrophilicity of OG-treated coal.

Mechanistically, surfactant modification enhances long-flame coal wettability through dual action: hydrophobic chains associate with native C–C/C–H domains, while hydrophilic headgroups (for example, carboxyl and hydroxyl) enrich the interface with oxygenated functionalities. The net result is increased surface polarity and higher water-adsorption energy, manifesting as substantially improved wetting and precise control over interfacial behavior.

#### 3.2.2. FTIR Analysis

**Figure 7:**
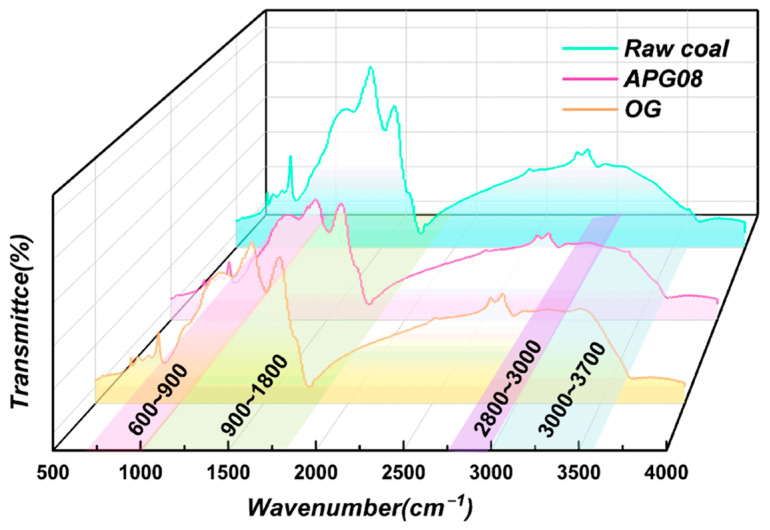
FTIR spectra of raw coal, APG08-modified coal and OG-modified coal.

To elucidate how surfactants regulate hydrophilic functionalities at the molecular-vibration level, we characterized surface groups in raw coal and in APG08- and OG-modified samples by Fourier-transform infrared spectroscopy (Figure 7), thereby complementing the microscale mechanism underlying enhanced wettability. Comparative survey spectra show four principal absorption regions: the hydroxyl band (3700–3000 cm^−1^), aliphatic C–H band (3000–2800 cm^−1^), oxygen-containing functional-group band (1800–900 cm^−1^) and aromatic band (900–600 cm^−1^) [52]. The overall line shapes remain unchanged after surfactant adsorption, indicating that while the relative abundances of functional groups shift, the fundamental group identities in the coal matrix are preserved.

A closer inspection of the spectra shows that the wavenumbers of the band maxima and the adjacent absorption minima for raw, APG08-modified and OG-modified coal are essentially identical within the instrumental resolution (±4 cm^−1^), and no systematic red- or blue-shift is observed among the three samples. The most significant differences between raw coal and modified coal concentrate are reflected in two wavenumber ranges related to hydrophilicity: the O–H stretching vibration window (3000–3700 cm^−1^) and the 900–1800 cm^−1^ region. Their positions of the main band maxima and the intervening minima coincide, whereas the depth and breadth of the bands differ. This behavior indicates that surfactant adsorption does not create new vibrational modes or fundamentally alter the type of surface functional groups; instead, it mainly changes their abundance and hydrogen-bonding environment. The deeper absorption features and broader envelopes observed for OG-modified coal are therefore attributed to a higher population of oxygen-containing groups and more strongly associated hydroxyls, rather than to a genuine spectral shift. Compared with branched APG08, the linear chain of OG imposes lower steric hindrance, enabling stronger adsorption on the coal surface and exerting a greater influence on hydrogen-bond formation. In addition, surfactant molecules associate with free surface hydroxyls via hydrogen bonding and, potentially, π–π interactions with aromatic domains, driving a transition from a “low-activity, free” state to a “high-activity, associated” O–H state (OH···OH). Consequently, OG-treated coal exhibits a higher population of associated hydroxyls and a richer complement of oxygenated groups, conferring stronger affinity for water and enabling multivalent, stable hydrogen bonding. These spectral signatures are consistent with the superior wettability observed for OG.

#### 3.2.3. SEM Analysis

**Figure 8:**
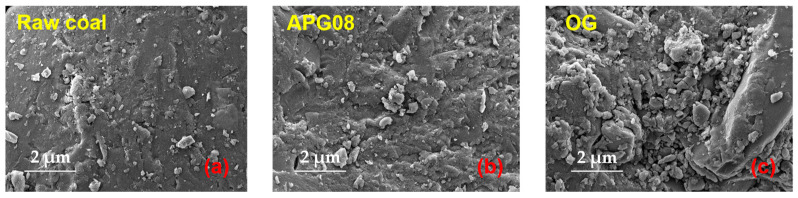
Structural characteristics of the surface morphology of the 3 coal samples.

Morphological features of raw and surfactant-treated coal were examined by scanning electron microscopy (SEM) at 1000× magnification. Representative micrographs for the three samples are shown in Figure 8. The raw coal exhibits pronounced, well-defined porosity with relatively dispersed surface particles and only minor, loosely bound agglomerates. By contrast, the modified samples display extensive adhesion of fine dust agglomerates; notably, OG-treated coal shows more numerous and larger aggregates, with broader spatial coverage, than APG08-treated coal. These aggregates—formed as surfactant-bound clusters of ultrafine particles—fill larger, multilayer pore structures and are not readily detached. Their infill subdivides macropores into a greater number of small pores, which enhances capillarity, facilitates complete penetration of water into surface porosity, and strengthens both water diffusivity and surface hydrophilicity [53]. Overall, the SEM observations indicate that pore filling arises not only from effective micelle adsorption within micropores but also from the formation of surfactant–fine-coal agglomerates that occupy larger pores.

### 3.3. Molecular Dynamics Simulation

#### 3.3.1. Contact Surface Area

**Figure 9:**
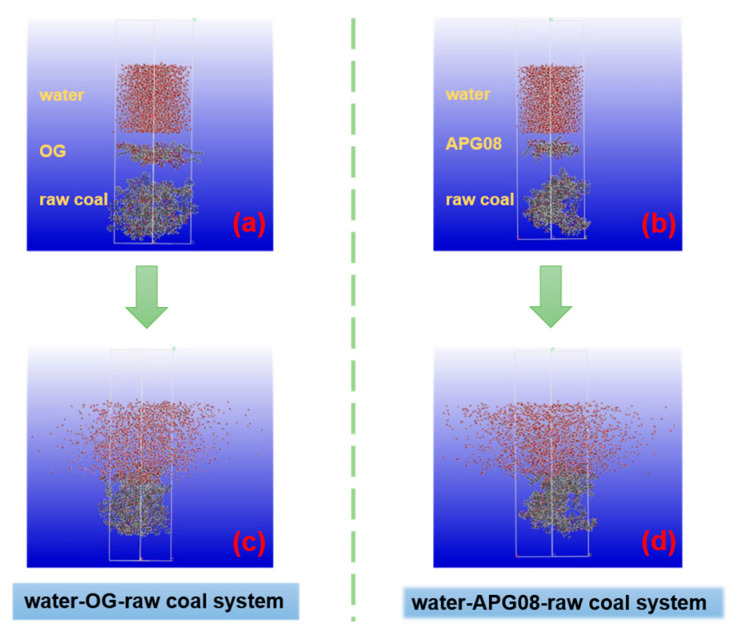
Molecular simulation process: (**a**,**b**) initial configurations of water-OG-raw coal system and water-APG08-raw coal system; (**c**,**d**) equilibrium configurations of water-OG-raw coal system and water-APG08-raw coal system.

Figure 9 shows the molecular simulation process. The contact surface area (CSA) reflects the adsorption strength between two materials and is positively correlated with it [54]. CSA was computed by:

(2) $$CSA=\left({SASA}_{anthracite}+{SASA}_{surfactant}-{SASA}_{total}\right)∕2$$

where SASA_anthracite_, SASA_surfactant_ and SASA_total_ are the solvent-accessible surface areas (SASA) of the long-flame coal model, the surfactant, and the combined coal–surfactant binary system, respectively. A probe radius of 0.14 nm was used. The CSA values for OG and APG08 are 2254.18 Å^2^ and 1072.23 Å^2^, respectively, indicating stronger adsorption of OG on long-flame coal. We attribute this enhancement to the denser interfacial network formed by OG on the coal surface.

#### 3.3.2. Mass Density Distribution

**Figure 10:**
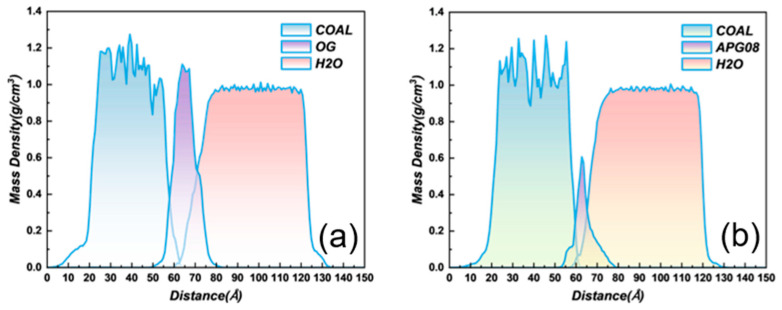
Mass density distribution of OG and APG08 modified coal: (**a**) OG modified coal mass density distribution; (**b**) APG08 modified coal mass density distribution.

Mass-density profiles along the *Z*-axis for each component are shown in Figure 10. For OG-modified coal, the overlap between the long-flame coal layer and the water layer spans 58.73–65.31 Å, with a separation of 6.58 Å and a peak density of 1.23 g cm^−3^. For APG08-modified coal, the overlap spans 57.47–61.96 Å, with a 4.49 Å separation and a peak density of 0.95 g cm^−3^. Thus, OG yields a broader interfacial overlap and a denser water domain at the interface, indicating a wider, higher-density network coverage on the coal surface [55]. The downstream shift of the water onset further suggests that OG assembles into a compact interfacial layer on the coal, mediating water–surface interactions while impeding direct contact; this is consistent with stronger adsorption of OG at the interface and superior wettability relative to APG08 [56].

#### 3.3.3. Interaction Energy

In the modified-coal systems, the interaction energy between the surfactant and long-flame coal serves as a proxy for adsorption strength: greater heat release (i.e., a more negative interaction energy) indicates a more stable post-adsorption configuration and stronger binding [57,58,59]. The total interaction energy, E, comprises van der Waals (EV) and electrostatic (EE) contributions and reflects the potential for wettability enhancement. These terms are defined as:

(3) $$EV={EV}_{total}-{EV}_{A}-{EV}_{B},$$

(4) $$EE={EE}_{total}-{EE}_{A}-{EE}_{B}$$

(5) E=EV+EE

Here, EE is the electrostatic interaction energy (kJ mol^−1^), EV is the van der Waals interaction energy (kJ mol^−1^), and E is the total interaction energy (kJ mol^−1^). EV_total_ and EE_total_ denote the total energies of the fully assembled system; EV_A_ and EE_A_ refer to the energies of the surfactant–coal adsorbate; EV_B_ and EE_B_ correspond to the energies of the water phase.

Interaction-energy results for OG and APG08 are summarized in Table 5. Comparison of E shows that the OG-modified system exhibits a more negative total interaction energy, implying greater energy release upon adsorption. This points to a more stable adsorption geometry and tighter interfacial binding of OG on the coal surface, consistent with enhanced surface hydrophilicity. Because the two surfactants share identical molecular weight and headgroup chemistry and differ only in alkyl-chain topology (linear for OG; branched for APG08), the superiority of OG is attributed to reduced steric hindrance in its linear chain, which permits a higher density of adsorbed oxygen-containing groups. This lower-hindrance configuration increases the population of hydrophilic sites at the interface and underlies the better wettability achieved with OG.

#### 3.3.4. Mean Square Displacement

To assess how the two surfactants modulate water accumulation at the coal interface, we computed the diffusion coefficient (*D*) of interfacial water from the mean-squared displacement (*MSD*) [60,61]:

(6) $$MSD=\frac{1}{N}\sum_{i=1}^{N}{\left[r_{i}\left(t\right)-r_{i}\left(0\right)\right]}^{2}$$

(7) $$D=\underset{t\to \infty }{\lim}\left(\frac{MSD}{6t}\right)=\frac{1}{6}K_{MSD}$$

where *MSD* is the mean-squared displacement (Å^2^), *N* is the number of tracked molecules, *D* is the diffusion coefficient (Å^2^ ps^−1^), *t* is time (ps), *r*(*t*) and *r*(0) are the position vectors at time *t* and 0, respectively, and *K_MSD_* is the slope of the *MSD*–time curve.

**Figure 11:**
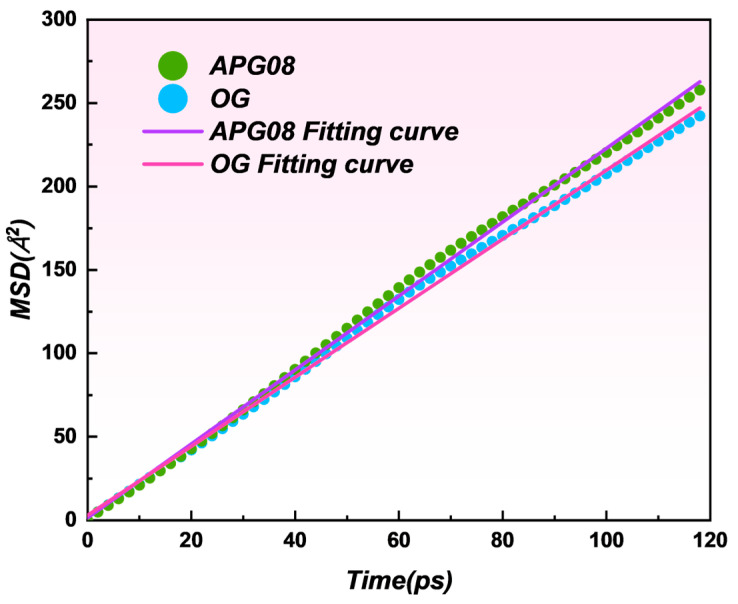
Mean square displacement of water molecules in OG and APG08 modified coal.

As shown in Figure 11, D equals 0.4135 Å^2^ ps^−1^ (R^2^ = 0.9981) in the OG system and 0.3264 Å^2^ ps^−1^ (R^2^ = 0.9989) in the APG08 system. OG forms a more stable adsorption layer on coal, fostering a denser hydrogen-bond network between the modified surface and adjacent water. The resulting increase in water–water cohesion reduces molecular mobility and lowers the diffusion rate, consistent with stronger wettability for OG-treated coal. By contrast, the APG08-modified surface supports fewer hydrogen bonds, weakens intermolecular attraction, and yields faster diffusion. Collectively, these results indicate that OG imparts greater hydrophilicity to the coal interface.

#### 3.3.5. Number of Hydrogen Bonds

Hydrogen bonds at the water/modified-coal interface were enumerated with a Perl script. Bonds were counted using a standard geometric criterion: donor–acceptor distance < 0.25 nm and H–acceptor angle > 135° [62]. The OG-modified system exhibits an average of 165 interfacial hydrogen bonds, versus 142 for APG08. These data reinforce the conclusion that OG establishes a denser hydrogen-bonding network at the coal surface. Enhanced accumulation of water molecules at the OG-treated interface underpins its superior wettability.

## 4. Conclusions

Experiments show that OG outperforms APG08 in wettability: it achieves a smaller contact angle (19.8°), a faster wetting rate (K = 0.4727) and a shorter sedimentation time (378 s). The linear alkyl chain of OG imposes less steric hindrance than the branched chain of APG08, enabling tighter, more ordered interfacial packing and greater adsorption stability, thereby yielding superior wetting.

FTIR, XPS and SEM analyses indicate that OG adsorbs more strongly on the long-flame coal surface. Its straight-chain alkyl groups effectively fill microscopic pores and promoting aggregation of ultrafine coal particles. This adsorption enriches hydrophilic and oxygen-containing functionalities, substantially increasing surface hydrophilicity and, in turn, wettability.

Molecular dynamics simulations reveal a cooperative mechanism in which π–π interactions between the glucose six-membered ring of OG and aromatic domains of the coal, together with the orderly, dense arrangement of hydrophobic segments and the formation of multilayer hydration networks, enhance interfacial binding. The resulting compact OG adlayer concentrates more water molecules near the modified surface, markedly reduces their mobility, and increases both adsorption and hydrogen-bond formation, thereby further improving the wettability of the treated coal.

## Figures and Tables

**Table 2:** Surfactant-related information.

Surfactant	Molecular Formula	Molecular Weight (g mol^−1^)	CMC (mg L^−1^)	Molecular Structure
OG	C_14_H_28_O_6_	292.37	1300	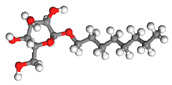
APG08	C_14_H_28_O_6_	292.37	1300	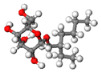

**Table 3:** Fitted parameters of the semi-empirical model for contact angle with time curves on long-flame coal.

Surfactant	*θ_i_* (°)	k (s^−n^)	n	R^2^
H_2_O	71.3	0.1447 ± 0.0147	0.4179	0.9587
APG08	49.2	0.2039 ± 0.0123	0.3239	0.9743
OG	36.5	0.4727 ± 0.0236	0.2093	0.9662

**Table 4:** C 1s and O 1s content of long-flame coal surface after adsorption.

	Functional Groups	Raw Coal			APG08			OG		
		Peak (BE)	FWHM (EV)	Atomic (%)	Peak (BE)	FWHM (EV)	Atomic (%)	Peak (BE)	FWHM (EV)	Atomic (%)
C1s	C–C/C–H(%)	284.80	1.51	76.21	284.80	1.56	73.60	284.80	1.59	70.44
	C–O(%)	286.44	1.48	12.04	286.50	1.55	18.19	286.54	1.33	19.62
	C=O(%)	288.11	3.37	7.16	288.21	1.91	6.07	288.01	1.96	8.09
	O=C–O(%)	290.46	3.27	4.59	290.33	1.91	2.14	290.37	1.96	1.65
O1s	C=O(%)	532.23	2.02	69.92	532.37	2.67	60.42	532.66	2.24	63.00
	C–O/OH(%)	533.54	1.61	26.79	533.14	1.74	35.37	533.15	1.53	29.85
	COO/COOH(%)	534.68	3.37	3.29	534.27	3.37	4.21	534.04	2.40	7.15

**Table 5:** The OG system and APG08 system interaction energy.

Model	EE (kcal mol^−1^)	EV (kcal mol^−1^)	E (kcal mol^−1^)
OG System	−1636	−347	−1983
APG08 System	−1275	−512	−1787

## Data Availability

The raw/processed data required to reproduce these findings cannot be shared at this time as the data also form part of an ongoing study.
